# Research productivity among Canadian first year dermatology residents: A 15 year analysis

**DOI:** 10.1016/j.jdin.2023.09.001

**Published:** 2023-09-09

**Authors:** Katrina D. Cirone, Mohamed Akrout, Daiana R. Pur, Ronald Vender

**Affiliations:** a Schulich School of Medicine and Dentistry, Western University, London, Ontario, Canada; b Department of Computer Science, University of Toronto, Toronto, Ontario, Canada; c Department of Medicine, McMaster University, Hamilton, Ontario, Canada; d Dermatrials Research Inc, Hamilton, Ontario, Canada

**Keywords:** authorship, bibliometrics, dermatology, medical education, resident productivity, resident selection

*To the Editor:* As matching into a Canadian dermatology residency program continues to increase in difficulty each year, evidenced by the declining match rate, it has become important to accurately characterize the scholarly profile of successful applicants.^1^ Although a variety of factors are considered during ranking, research publications are often used as a proxy for academic potential and can be considered a valuable, objective, and quantifiable variable which can demonstrate academic abilities and work ethic, indicate interest in the specialty, and allow students to begin developing relationships with practicing dermatologists.^2^

Research can substantially affect the chance of a successful match and remains one of the few quantifiable metrics that can be controlled and modified by applicants.^3^ As such, applicants are under pressure to generate research publications to strengthen their application. This retrospective database study quantitatively characterizes trends in research productivity among medical students who matched into Canadian dermatology residency programs over the past 15 years.

A review of national and provincial physician databases (Royal College of Physicians and Surgeons of Canada and College of Physicians and Surgeons of Ontario), was conducted to identify successful dermatology applicants that began their first year of training between 2008 and 2022. Metrics reflective of research productivity at the tie of residency application (publication count, dermatology publications, authorship position, and H-index) were obtained from Scopus and trends were identified and evaluated. Successful applicants were divided into 3 cohorts covering the 3 time periods 2008-2012, 2013-2017, and 2018-2022.

From the 10 Canadian residency programs, the 371 incoming dermatology applicants generated 828 publications (mean 2.23 ± 2.07; median 1), of which 329 were dermatology-related (mean 0.89 ± 1.79; median 0), and the mean H-index was 1.36 ± 1.49. A significant increase (*P* < .001) in all research productivity metrics was observed during 2018-2022 compared with 2008-2012 and 2013-2017 (Fig 1 and Table I). Our results have determined that over the past 15 years, the amount of applicants that matched into dermatology residency programs with at least 1 publication has almost doubled, the total number of publications tripled, and the total number of dermatology publications increased 7-fold; which is twice the rate as nondermatology publications. Further, the significant increase in H-index and first author publications, despite the decreased time since publication, indicates the scholarly impact of publications continues to grow, and may suggest students are taking on greater leadership and ownership of projects.

**Fig 1:**
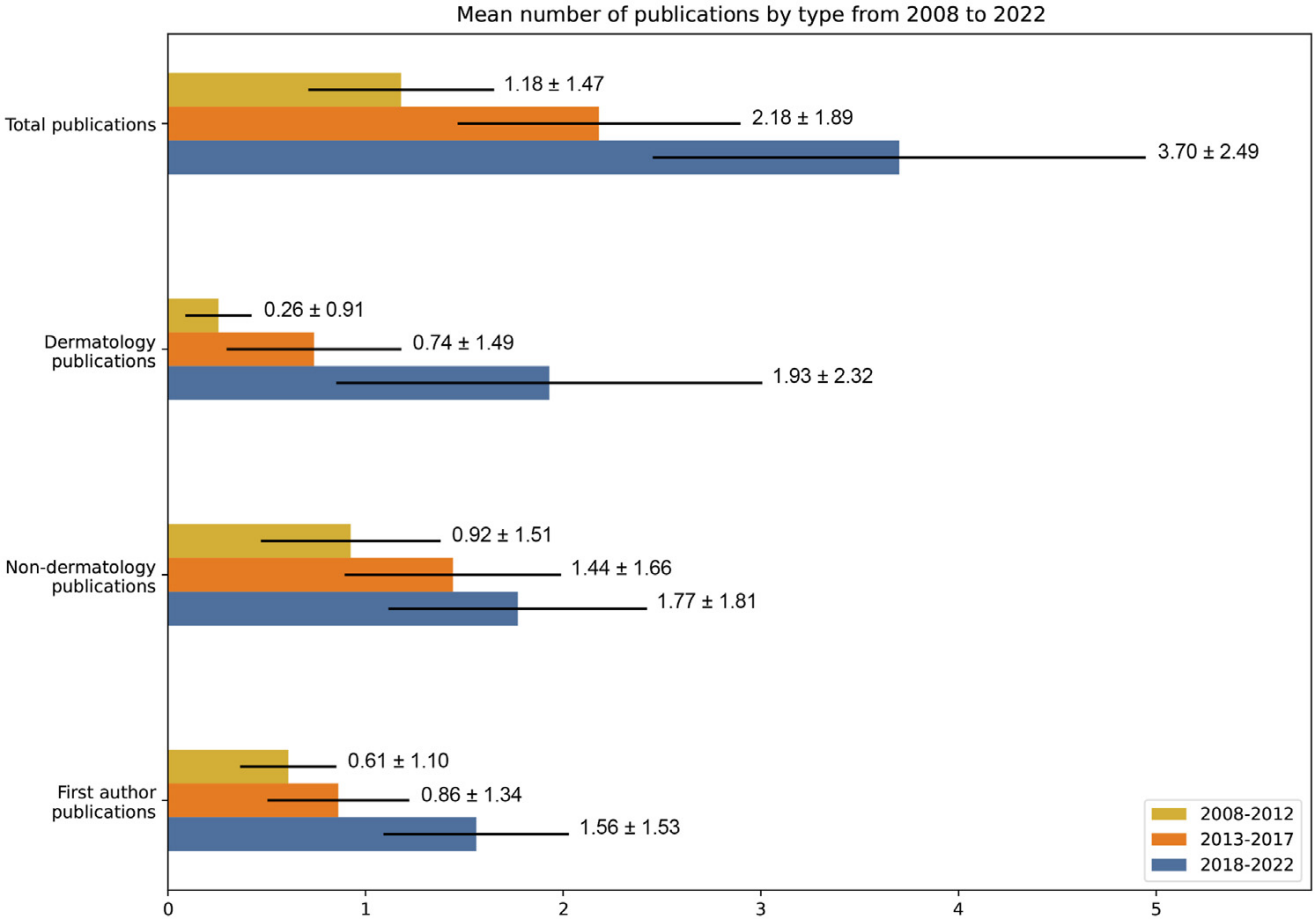
The mean number of research publications by type divided into cohorts covering the 3 time periods 2008-2012, 2013-2017, and 2018-2022 (mean ± SD).

**Table I:** Descriptive statistics of research productivity and research impact of successful dermatology applicants from 2008 to 2022 by year and type of publication

Metric	2008	2009	2010	2011	2012	2013	2014	2015	2016	2017	2018	2019	2020	2021	2022	Total
H-index	0.60 (1.02)	0.84 (1.44)	1.21 (1.47)	1.00 (1.42)	1.22 (1.68)	0.82 (1.17)	1.36 (2.56)	1.87 (1.60)	2.17 (1.83)	1.64 (1.47)	1.95 (1.61)	1.41 (1.17)	1.32 (1.16)	1.82 (1.57)	1.36 (1.21)	1.36 (1.49)
Total publications	0.72 (1.07)	0.96 (1.49)	1.39 (1.50)	1.44 (1.51)	1.35 (1.76)	1.03 (1.23)	1.76 (3.05)	2.13 (1.66)	3.38 (2.31)	2.71 (1.98)	3.25 (1.99)	3.76 (1.93)	2.32 (1.40)	3.86 (2.52)	5.09 (3.26)	2.23 (2.07)
Dermatology publications	0.24 (0.72)	0 (0)	0.32 (0.97)	0.33 (0.83)	0.35 (1.10)	0.33 (0.96)	0.60 (0.96)	0.39 (0.85)	1.72 (2.06)	0.61 (1.28)	1.25 (1.48)	2.29 (1.87)	0.95 (1.21)	1.45 (1.81)	3.59 (3.22)	0.89 (1.79)
Publications as first author	0.44 (0.91)	0.28 (0.86)	0.82 (1.28)	1.00 (1.17)	0.59 (1.12)	0.39 (0.98)	0.48 (0.82)	1.17 (1.24)	1.52 (1.74)	0.82 (1.24)	1.65 (1.49)	2.00 (1.61)	0.84 (0.91)	1.32 (1.42)	2.00 (1.82)	0.96 (1.35)

Values listed as mean (standard deviation).

There appears to be a greater scholarly focus from residency programs and trainees as indicated by the increased research output among medical students that successfully matched into Canadian dermatology residency programs, seen in the setting of increased match competition. This may suggest an increased emphasis placed on medical research by both students and residency programs, a finding that has also been described in the United States and other competitive specialties throughout Canada.^4^ Future studies can assess whether research productivity at the time of match is predictive of research output during residency, choice of pursuing a fellowship, or practice setting.

## Conflicts of interest

None disclosed.
